# Supplementary material for: Dietary assessment and prevention of hypertension in Nigeria: Protocol for a retrospective cross-sectional study for the development and validation of a food frequency questionnaire for clinical use
Source: PLoS One. 2024 Apr 17;19(4):e0292561. doi: 10.1371/journal.pone.0292561 (PMC11023198; doi:10.1371/journal.pone.0292561)
Supplement: S1 Protocol — (PDF) [file pone.0292561.s001.pdf]

UNIVERSITY OF LEEDS

Main Form

THIS ONLINE ETHICS REVIEW SYSTEM IS BEING PILOTED AND IS ONLY AVAILABLE FOR APPLICANTS FROM THE FACULTIES OF BUSINESS, ENVIRONMENT AND SOCIAL SCIENCES REVIEWED BY THE AREA FREC and submitted on or after Monday 4th July 2022 -any queries, please email EthicsEnquiries@leeds.ac.uk

What is the title of the research? (Project title)

Dietary Assessment and Prevention of Hypertension in Nigeria

Chief Investigator

Title

First Name

Nimisoere

Surname

Batubo

Email

fsnpb@leeds.ac.uk

Which Faculty do you belong to?

Business, Earth & Environment, Social Sciences (AREA FREC)

Which School/Institute/Dept (or other) are you in?

Food Science and Nutrition

Will the research be conducted using already published data or data in the public domain?

See here for guidance on already published data or data in the public domain.

- [ ] Yes
- [x] No

Will the project involve the NHS?

- [ ] Yes
- [x] No

Please answer the following questions:

- [ ] I am a substantive employee
- [ ] I am an undergraduate student
- [x] I am a PGR
- [ ] I am a taught PG
- [ ] I am visiting or honorary staff

NOTE:

Your academic supervisor must activate their account via ResearchEthics.leeds.ac.uk and then log out. You will then be able to select them from the search box in this form to enter their details and request their signature declaration so you can submit

Intended study start date?

01/05/2023

Intended fieldwork start date:

01/06/2023

Intended fieldwork end date:

20/12/2024

Intended study end date:

30/04/2024

Is this review request time-sensitive, e.g. due to funder's deadlines?

- ☐ Yes
- ☑ No

Are there other members included in the research team?

- ☐ Yes
- ☑ No

Will the research team include an academic supervisor?

- ☑ Yes
- ☐ No

Academic supervisor

Title

Dr

First Name

Michael

Surname

Zulyniak

Email

M.A.Zulyniak@leeds.ac.uk

What is the purpose of the application?

- ☐ Research
- ☐ Research involving animals

  To check if any other relevant approvals are required please email : h.o.admin@leeds.ac.uk

- ☐ Educational qualification
- ☑ Educational Research & Evaluation
- ☐ Medical Audit or Health Service Evaluation
- ☐ Module Block Approval for Taught Students

  see here for guidance on block approvals

- ☐ Proportionate Review
- ☐ Other

Please select all that apply to describe the research from the list below:

- ☐ Research which has potential adverse environmental impact.

  Environmental impact guidance info

- ☐ Research working with data of human participants
- ☑ Research on or with human participants
- ☐ Research working with human tissue samples

Will informed consent be provided by participants?

- ☑ Yes
- ☐ No

Upload Participant Information Sheet and consent form (template can be downloaded here) or a copy of informed consent statement if using anonymous online survey (see here)

Documents

Type	Document Name	File Name	Version Date	Version	Size
Informed Consent / Participant Information Sheet	Participant_Information_Sheet	Participant_Information_Sheet.doc	18/03/2023	1	88.5 KB
Informed Consent / Participant Information Sheet	Participant_Consent_Form	Participant_Consent_Form.doc	18/03/2023	1	80.5 KB
Informed Consent / Participant Information Sheet	Participant_Information_Sheet_v2	Participant_Information_Sheet_v2.doc	18/04/2023	2	96.5 KB
Informed Consent / Participant Information Sheet	Participant_Consent_Form_v2	Participant_Consent_Form_v2.doc	18/04/2023	2	83.5 KB

Research on or with human participants: Please select any relevant groups from list below:

- ☐ Children under 16
- ☐ Adults with learning disabilities
- ☐ Adults with other forms of mental incapacity or mental illness
- ☐ Adults in emergency situations
- ☐ Prisoners or young offenders

  See here and here for guidance

- ☐ Those who could be considered to have a dependent relationship with the researcher

  e.g colleagues - see here for guidance

  and e.g students - see here for guidance

- ☐ Other 'vulnerable' groups
- ☑ None of the above

Does the research involve external funding?

- ☑ Yes
- ☐ No

Funding Source

Tertiary Education Trust Fund

Funding Reference

TetFund

Grant Holder

Nimisoere Batubo

Project Ethical Risks

Please indicate any/all of the following that applies to the project:

- ☐ Any elements of deception?
- ☐ Is it possible that any criminal or other disclosures requiring action (i.e. safeguarding) might be raised?
- ☐ Discussion of topics that could be considered sensitive, embarrassing or upsetting (e.g. relationships, sexual activity, drug use, volunteered medical information?)
- ☐ The study will involve intrusive interventions or data collection methods (e.g. the administration of substances; involving physical or emotional stress; potential for harm
- ☐ The study will involve prolonged or repetitive testing
- ☐ The study will involve participatory action research or members of the public in a research capacity
- ☐ Participants will be taking part in the research without their knowledge and consent (e.g. covert observation of people in non-public places)
- ☐ The study will involve social media and participants recruited or identified through the internet.

  See here for guidance

- ☐ Financial inducements (other than reasonable expenses and compensation for time) will be offered to participants.

  See here for guidance

- ☐ Risks to the safety and wellbeing of the researchers, or individuals not directly involved in the research?
- ☐ The study will involve international partners/collaborators or research undertaken outside the European Economic Area where there may be issues of local practice and political sensitivities
- ☑ The study requires in-country ethics approval or permissions (these could be applied for in parallel to University of Leeds ethics approval)*
- ☐ The study will involve anonymised secondary data from an external party
- ☐ The study will involve linking or sharing of data or confidential information beyond the initial consent
- ☐ The study will involve the transfer of identifiable data outside the European Economic Area
- ☐ The study will require DBS checks

  See here for guidance (required for research with children or 'vulnerable' adults)

- ☐ The study will require local managerial or gatekeeper permissions for access to potential participant groups (e.g. Head of School to access school pupils; organisational permission to access staff; University of Leeds permission to access students or staff, including MBChB* gatekeeper permission to access University of Leeds Medical Students)
- ☐ None of the above apply

Please provide details of any ethical issues indicated above and explain how these will be addressed

None

Please describe any/all potential benefits and risks to participants in the short and medium terms?

• Increased self-awareness: Completing the Food Frequency Questionnaire (FFQ) may help you become more aware of your own dietary habits and provide you with a better understanding of the foods and beverages you consume on a regular basis.
• Contribution to scientific research: Your participation in the study will contribute to the our understanding of the dietary habits of adult sliving in Nigeria and its link with hypertension.
• Improved health outcomes: The information gathered in the study will help in the development of a clinical practical nutritional assessment tools for dietary assessment in Nigeria and the prevention of hypertension.
• Personal satisfaction: Participating in the study may provide a sense of personal satisfaction and pride in contributing to scientific research and improving public health outcomes.

Summary of Research

Please provide a short summary of the research using language easily understood to the lay person, and cover the main parts of the study proposal/protocol, specifically:

- **The background of the research and why it is important**
- **The questions it will answer and potential benefits**
- **The study design and what is involved for participants**

Hypertension (i.e., high blood pressure) is a major global public health concern and a major modifiable risk factor for cardiovascular diseases (CVD), premature death, stroke, chronic kidney disease, and dementia. Globally, around 1/3 of all women and men (~1.3 billion adults) in the world have hypertension, but in low- and middle-income countries, including Africa, it can affect almost 50% of adults. Also, while the number of people with hypertension in the UK, the US, and China has gone down over the past decade, in Nigeria and other West African reporting, it has increased by ~15%.

We know that hypertension is linked to unhealthy diets and lack of physical activity. For this reason, Nigeria and other West African countries outlined national nutritional guidelines to combat hypertension. However, without nutritional tools to evaluate their patients' diet, clinicians have struggled to (i) discuss and prescribe healthy eating with their patients and (ii) make a real change in eating habits and hypertension. To address this, the project aims to design a nutritional assessment tool to improve the level of nutritional support and guidance available to Nigerian clinicians so they can provide personalised nutritional guidance to patients to combat hypertension.

The estimated duration of the project is 12 months. The project will be divided into 4 phases. Questionnaire design and recruitment (Phase 1), Feedback study (Phase 2), Reliability and validity study (Phase 3), and Feasibility and acceptability study (Phase 4) are estimated to last three (3) months each. Participation in the study will involve completing a questionnaire and physiological measures (blood pressure, height, and weight) collected in phases 2,3 and 4 (i.e., a maximum of 3 times). Additionally, two weeks after completing the FFQ, participants will be asked to complete three 24-hour recall questionnaires in phase 3 and be asked to provide their thoughts and feedback on the FFQ. The results of the feasibility and acceptability study will be used to refine the FFQ and prepare for a larger study in the future.

Please identify any ethical considerations or issues with the research and clearly state how these will be addressed

Some of the ethical considerations that have been identified include confidentiality and privacy, bias, inaccuracy, and voluntary participation. To address these ethical considerations, researchers should:

1. To ensure that participant information is kept confidential and secure, all paper questionnaires will be stored securely under lock and key in a locked cabinet.
2. When the data is transferred to a digital spreadsheet, it will be password protected and stored on a 256-bit encrypted university drive.
3. For accessibility, all questionnaires will be written and presented at a level that is understandable to the majority of the public.
4. Individuals will be encouraged to take as much time as needed to complete the questionnaires and reminded that they are not obligated to answer any questions that make them uncomfortable.
5. Informed consent will be collected from all participants.
6. Participants will be informed that they are free to withdraw at any time without impacting their access to health care or support.

What will the researcher do if a participant is found to have high blood pressure that they were unaware of as part of this screening? Will they be informed of this and directed toward relevant health care services or advice?

If a participant is found to have high blood pressure during the study screening that was previously unknown to them, the principal researcher will inform the participant of the finding and provide guidance on how to seek appropriate medical care. This may involve referring the participant to a healthcare professional or providing them with information on how to access relevant healthcare services in the study location (RSUTH).

What the aims of the study (must be comprehensible to a lay person)

The project aims to design nutritional assessment tools that would be used to improve the level of nutritional support available to Nigerian clinicians so they can provide personalised nutritional guidance to patients to combat hypertension. Study 1 will test the suitability of the diet screening tool for clinicians and patients in Nigeria, while Study 2 will test its ability to predict people at elevated risk of high blood pressure.

Please describe the methodology of the research

The study will be conducted in 4 phases:
(1) Phase 1: Development of a short food frequency questionnaire (FFQ) from the food list, which is informed by the results of the systematic review and meta-analysis, West Africa food composition table and the Nigeria National Nutritional Guidelines for Non-communicable diseases and the recruitment of study participants.
(2) Phase 2: Feedback study of the FFQ (n=50)
(3) Phase 3: A feasibility and validity of the FFQ against three 24-hour dietary recalls (n=50).
(4) Phase 4: The assessment of the feasibility and acceptability of the FFQ (n=150).

Please describe specifically what participants will be asked to do in the study (i.e. number of visits, activity undertaken during visit, time / travel required, interviews, survey etc)

The participants will be asked to complete a 33-item FFQ (see Short_Form_Food_Questionnaire.docx) once during the visit in phases 2, 3 and 4 that asks about their dietary habits and lifestyle habits, socio-demographic characteristics and the measurement of their blood pressure, height and weight once during each visit in phases 2-4. Additionally, the participants will be asked to complete three 24-hour recall questionnaires in phase 3 only two weeks after completing the refined FFQ.

Where will the data collection be undertaken? If off campus, a fieldwork risk assessment is usually required – see here for guidance

Data will be collected in Rivers State University Teaching Hospital (RSUTH), Port Harcourt, Rivers State, Nigeria.

Upload any required risk assessment here:

Please note: Risk assessments are a University requirement for all fieldwork taking place off campus. The risk assessment forms and further guidance on planning for fieldwork in can be found on the Health and Safety section of the Research Ethics website University's Health & Safety website, along with further information about risk assessment.

Documents

Type	Document Name	File Name	Version Date	Version	Size
Risk Assessment Form	Risk_Assessment	Risk_Assessment.docx	16/03/2023	1	142.5 KB
Risk Assessment Form	Risk Assessment_v2	Risk Assessment_v2.docx	19/04/2023	2	150.7 KB

Recruitment & Informed Consent Process

How will potential participants (Please describe in detail):

Be identified?

Participants for studies 2, 3 and 4 will be identified in the hospital or outpatient clinics by filling out a short questionnaire for participant selection. They will include adults aged 18 to 70 years, without a diagnosis of hypertension, that are residents of Port Harcourt, Rivers State, Nigeria. This will require ethical approval from the Rivers State Ministry of Health, Rivers State University Teaching Hospital.

Be approached regarding their potential participation?

Potential participants will be approached through their healthcare providers, such as doctors, nurses, or dieticians, who can refer their patients to the study. Researchers may also set up informational tables in high-traffic areas within the hospital or clinic, such as the waiting room, to recruit potential participants using recruitment posters/questionnaires.

Be recruited to the study?

Once identified, potential participants will be contacted by study personnel, have the study discussed with them, and be provided with a Participant Information Sheet (PIS) to read and be given an opportunity to ask any questions. If they express an interest in participating, they will be provided informed consent before the commencement of the study.

Undergo the informed consent process and be informed of exactly what is required of them if they agree to participate?

A link to the Research Participant Privacy Notice must be provided in the participant information about the study.

All recruited participants will undergo the informed consent process. This will help ensure that participants are fully informed and able to make an informed decision about whether to participate in the study. A copy of the signed informed consent and the participant's information sheet will be made available for them. Once enrolled, if requested, they may request any data collected on them or limit/refuse its use for any unpublished research.

If you intend not to receive informed consent, please provide a rationale as to why this is the case below or state not-applicable

N/A

Will you be excluding any groups of people and if so, please provide a clear rationale for that?

Certain groups of people will be excluded from participating in this study:

Pregnant women: Pregnant women may be excluded from the study due to the potential changes in their diet and nutritional status during pregnancy, which may affect the accuracy of the FFQ. Additionally, some of the questions in the FFQ may pertain to food items or supplements that are not recommended for pregnant women.

Children: Children under 18 years will be excluded from the study due to the potential for changes in their diet and nutritional status during growth and development, which may affect the accuracy of the FFQ.

People with severe medical conditions: People with severe medical conditions, such as liver, cancer, endocrine diseases, diabetes or kidney disease, will be excluded from the study due to the potential for changes in their diet and nutritional status related to their condition, which may affect the accuracy of the FFQ.

People with current treatment for hypertension: People currently undergoing treatment for hypertension may be excluded from the study, as the treatment they are receiving may affect their dietary habits and the accuracy of the FFQ.

These exclusions are based on the rationale that the information collected from these groups of people may not accurately reflect their typical dietary habits and may not provide meaningful data for the development of the FFQ. Excluding these groups of people from the study can help ensure that the results are representative and accurate.

How many participants do you envisage to be required to meet the research aims and objectives? If you have a formal power calculation, please provide it here

Phase 2 and 3:
With a sample size of 50, we will be able to estimate a drop-out rate of 80% to within a 95% confidence interval of +/- 11%, which will allow us to support any necessary amendments for STUDY 2 recruitment time based on local data (National Institute for Health Research). Additionally, with more than 30 individuals, we will be well positioned to achieve saturation for feasibility goals: Screening rate, Screening proportion, Eligibility proportion, Exclusion Reasons, Consent rate, and Completion rate.

STUDY 4:
The correlation coefficients between FFQ nutrient intake estimates and reference instruments from previous studies range from 0.4–0.6 have been reported (Willett, 2013). The sample size was calculated from the correlation coefficient (0.4-0.6) using the following formula:

n = (Zα +Zβ)^2 x σ^2/d^2

Where n= sample size
Zα = critical value of the standard normal distribution for the specified alpha level= 1.96
Zβ = critical value of the standard normal distribution for the specified power= 0.84
σ = common standard deviation of the two variables= 0.5
d = difference between the expected and null hypothesis correlation coefficients.

The sample size to detect a correlation coefficient of 0.5 between the FFQ nutrient intake estimates and reference instruments, assuming a two-tailed alpha level of 0.05, a power of 0.80, a conservative estimate of the common standard deviation of 0.5, and critical values of 1.96 and 0.84, respectively, as follows:

n = (1.96 + 0.84)2 x 0.52 / (0.5 - 0.5)2
n = 102.4

Therefore, a minimum sample size of 103 participants would be required. Considering missing/ incomplete data and other study characteristics, the estimated sample size would be 150 participants.

Will participants be able to withdraw from the study?

- ☑ Yes
- ☐ No

Please state at which point in the data collection / research process this would be possible and state any identified time point (i.e. 10 days after an interview)

Participants can withdraw at any time and have their data removed from any unpublished research.

How long will participants have to decide their participation in the research? Please provide a rationale on the timeframe

Participants requiring additional time before providing informed consent will be offered our contact details and informed of their need to decide ahead of the study end date. They will also be asked if they would like us to contact them via email or phone as a friendly follow-up. If so, we will collect this information and contact them as requested up to 3 times over the next week. If we cannot reach them or they request that we not call back, we will not contact them again. If they contact us, we will re-attempt to contact them 1 more time. We will continue this until the study's end date.

Are there any arrangements for participants who may have challenges understanding verbal or written information, or whom have particular communication needs that could be addressed to facilitate their involvement in the research?

Participants with challenges in understanding verbal or written information will be supported by their caregivers and family members and also be provided with a food photo album.

Research Data Management & Storage

Do you have a data management plan?

See here for guidance

- [x] Yes
- [ ] No

Will you be processing, or have access to, any personal identifiable data during any stage of the study?

See here for guidance

- [x] Yes
- [ ] No

What is the data source?

- [x] New data collected for this research
- [ ] Data previously collected for other research
- [ ] Data previously collected for non-research purposes
- [ ] Data already in the public domain
- [ ] Other

How will the data be collected?

- [ ] Through one-to-one research interviews
- [ ] Through focus groups
- [x] Self-completion (eg questionnaires, diaries)
- [ ] Through observation
- [ ] Through autoethnographic research
- [ ] Through experiments/ user-testing involving participants
- [ ] From external research collaborators
- [ ] Other

Explain what measures will be put in place to protect personal data. E.g. anonymisation procedures, secure storage and coding of data. Any potential for re-identification should be made clear to participants in advance.

See here and here for guidance.

Individual potential participants will be allocated a unique study ID, and all data will be recorded with reference to this. The data will therefore be coded and anonymised. All electronic data will be password protected and only accessible to the PI and hard copies will be stored in locked metal filing cabinets accessible only by the site PI. A project management database will be located on a single server at the University of Leeds that is protected from unauthorised access by: (i) Logical security — firewall, antivirus, daily application of patches/updates, off-site encrypted backups – with access to the server only possible via an SSL connection; and (ii) Physical security – only authorised persons allowed access, use of hard disk drives configured for redundancy, use of an uninterruptible power supply, sited in a fire alarmed room. All data will be securely stored (256-bit encryption) by the University of Leeds' Research and Innovation Service on a Dell Compellent Storage array with multiple tiers of disks and RAID levels (RAID 10, RAID 5-9, RAID 6-10). For backup, two copies of the data are on site that is in different Data Centres, and every four hours, the data is copied over to an Offsite location. The data is protected by Checkpoints which enables backups for up to 12 months.

How will you make your research data available to others in line with the University's, funding bodies' and publishers' policies on making the results of publicly funded research publicly available. Explain the extent to which anonymity will be maintained.

See here and here for guidance.

Identifiable or personal contact information data will never be suitable for sharing but will be held securely until study completion. Anonymised data will be stored in the institutional system on publication but probably before and in whatever method the publisher may require, available on request. It is likely that requests for access to this data (anonymised) will be occasional, with few issues to prevent sharing. Therefore, the PI will formally review and discuss access requests for proposals. All significant decisions (approval, referral back for further information, and decline) will be documented for subsequent independent review. Independent of the study, an advisor with appropriate expertise is appointed to periodically review the outcomes of access requests post hoc. The reviewer may also be tasked to advise on a study's access policy and procedures.

Will the research involve any of the following activities at any stage (including identification of potential research participants)?

Will the research involve any of the following activities at any stage (including identification of potential research participants)?

- [ ] Examination of personal records by those who would not normally have access
- [ ] Access to research data on individuals by people from outside the research team
- [ ] Electronic surveys
- [x] Other electronic transfer of data
- [x] Use of personal addresses, postcodes, faxes, e-mails or telephone numbers
- [ ] Use of audio/ visual recording devices
- [ ] FLASH memory or other portable storage devices
- [x] Storage of personal data on, or including, any of the following:
    - University approved cloud computing services
    - Other cloud computing services
    - Manual files
    - Private company computers
    - Laptop computers
    - Home or other personal computers (data should be stored on a University of Leeds server such as your M: or N: drive or Office365 where it is secure and backed up regularly. See here for guidance)

Please specify details of mode of data transfer

Participants' data will be securely transferred over the University of Leeds 128-bit encrypted cloud storage from Port Harcourt, Nigeria and the University of Leeds.

How do you intend to report and disseminate the results of the study?

See here for guidance.

- [x] Conference presentation
- [x] Peer reviewed journals
- [x] Publication as an eThesis in the Institutional repository
- [ ] Publication on website
- [ ] Other publication or report
- [x] Submission to regulatory authorities
- [ ] Other
- [ ] No plans to report or disseminate the results

For how long will data from the study be stored?

See the UKRI Common Principles on Data Policy and here for guidance.

Students: It would be reasonable to retain data for at least 2 years after publication or three years after the end of data collection, whichever is longer.

Years 5

Months 0

Please explain why this length of time has been chosen

This period of time is sufficient to allow for follow-up research or analysis, demonstrates compliance with ethical and data protection regulations, and ensures the accessibility of the data for a sufficient period of time.

University Policy and Protocol Compliance

Information

Please be aware that by agreeing to the statements below, you are confirming that you have read and have understood the relevant University policies and or protocols. It will be evident in your ethics application if this is not the case and if so, your submission may be rejected.

Is a risk assessment required, including for fieldwork or lone working?

See here and here for guidance.

☐ Yes ☑ No

I confirm that I have read and understood the current version of the University of Leeds Research Ethics Policy.

The Policy is available here

☑ Yes ☐ No

I confirm that I have read and understood the current version of the University of Leeds Research Data Management Policy.

The policy is available here

☑ Yes ☐ No

I confirm that research participants will be provided with a copy of the Privacy Notice for Research.

Guidance is available here

☑ Yes ☐ No ☐ Not Applicable

I confirm that I have read and understood the current version of the University of Leeds Information Protection Policy.

The policy is available here

- [x] Yes
- [ ] No

Please indicate with which of the following UoL Protocols your study will comply?

	Yes	No	Not Applicable
Protocol on the protection, anonymisation and sharing of research data	[x]	[ ]	[ ]
Informed consent protocol	[x]	[ ]	[ ]
Verbal consent protocol	[ ]	[ ]	[x]
Protocol on the reimbursement of research participants	[ ]	[ ]	[x]
Low risk observation protocol	[x]	[ ]	[ ]

Conflicts of Interest

Will any of the research team or their institutions receive any other benefits or incentives for taking part if this research, over and above their normal salary and/or costs of undertaking the research?

- [ ] Yes
- [x] No

Is there scope for any other conflict of interest? See here for guidance

- [ ] Yes
- [x] No

Will the research funder have control of publication of research findings?

- [ ] Yes
- [x] No
- [ ] Not funded

Sharing information for training purposes

Are you content for information in the application to be used for research ethics and research data management training purposes within the University of Leeds. All personal identifiers and references to researchers, funders and research units would be removed.

- [x] Yes
- [ ] No

Supporting Documents

Supporting Documents

- [x] Recruitment materials

  e.g. poster, email text or social media text to be used to invite people to participate in your research project or advertise your study.

- [ ] Letter/email evidencing permission from host/gatekeeper if accessing for recruitment purposes
- [ ] Evidence of managerial permission

  i.e. From Head of Department or School or Line Manager if recruiting staff from the University, or from an external company or organisation

- [x] Questionnaire/survey questions and/or interview topic guide
- [x] Data management plan

  see the Collecting Research Data webpage and https://library.leeds.ac.uk/info/14062/research_data_management/62/data_management_planning

- [ ] Other

  e.g. data processing agreement (if personal data being transferred to 3rd party), transcription confidentiality agreement (if internal University staff transcriber is used)

Recruitment materials:

e.g. poster, email text or social media text to be used to invite people to participate in your research project or advertise your study.

Documents

Type	Document Name	File Name	Version Date	Version	Size
Recruitment Materials	Rrecuitment_poster_clinicians	Rrecuitment_poster_clinicians.pdf	18/03/2023	1	438.3 KB
Recruitment Materials	participant poster	participant poster.pdf	18/03/2023	1	298.3 KB
Recruitment Materials	participant poster	participant poster.pdf	17/04/2023	2	350.5 KB
Recruitment Materials	Rrecuitment_poster_clinicians	Rrecuitment_poster_clinicians.pdf	17/04/2023	2	441.6 KB

Questionnaire/survey questions and/or interview topic guide

Documents

Type	Document Name	File Name	Version Date	Version	Size
Questionnaire/survey and/or interview guide	Short_Form_Food_Questionnaire	Short_Form_Food_Questionnaire.docx	18/03/2023	1	122.9 KB

A data management plan (if applicable)

see the Collecting Research Data webpage and https://library.leeds.ac.uk/info/14062/research_data_management/62/data_management_planning

Documents

Type	Document Name	File Name	Version Date	Version	Size
Data Management Plan	Data_management_plan_v2	Data_management_plan_v2.pdf	21/03/2023	1	92.2 KB

Declarations & Signatures

Declarations

- ☑ The information in this form is accurate to the best of my knowledge and belief and I take full responsibility for it.
- ☑ I undertake to abide by the policies and guidelines stated below, and the ethical principles underlying good practice guidelines appropriate to my discipline.

  University's ethical and https://wsh.leeds.ac.uk/safety-topics policies and guidelines

- ☑ If the research is approved I undertake to adhere to the study protocol, the terms of this application and any conditions set out by the Research Ethics Committee.
- ☑ I undertake to ensure that all members of the research team are aware of the ethical issues and the contents of this application form.
- ☑ I undertake to seek an ethical opinion from the REC before implementing any amendments to the protocol.

  Guidance on amendments

- ☑ I undertake to submit progress/end of project reports if required.
- ☑ I am aware of my responsibility to be up to date and comply with the requirements of the law and relevant guidelines relating to security and confidentiality of personal data.
- ☑ I understand that research records/ data may be subject to inspection for audit purposes if required in future.

  Guidance on auditing

- ☑ I understand that personal data about me as a researcher in this application will be held by the relevant FRECs and that this will be managed according to the principles established in the Data Protection Act 2018 and the principles of the GDPR.

  Guidance on Data Protection Act 2018 and GDPR

Applicant's Signature

Signed: This form was signed by Nimisoere Batubo (fsnpb@leeds.ac.uk) on 19/04/2023 9:36 AM

Supervisor's Signature

Signed: This form was signed by Michael Zulyniak (M.A.Zulyniak@leeds.ac.uk) on 02/05/2023 6:51 PM
